# Radix paeoniae alba polysaccharide attenuates lipopolysaccharide-induced intestinal injury by regulating gut microbiota

**DOI:** 10.3389/fmicb.2022.1064657

**Published:** 2023-01-12

**Authors:** Aoyun Li, Jinxue Ding, Ting Shen, Ying Liang, Fan Wei, Yi Wu, Mudassar Iqbal, Muhammad Fakhar-e-Alam Kulyar, Kun Li, Kunhua Wei

**Affiliations:** ^1^College of Veterinary Medicine, Institute of Traditional Chinese Veterinary Medicine, Nanjing Agricultural University, Nanjing, China; ^2^College of Veterinary Medicine, Huazhong Agricultural University, Wuhan, China; ^3^Guangxi Key Laboratory of Medicinal Resources Protection and Genetic Improvement, Guangxi Engineering Research Center of TCM Resource Intelligent Creation, Guangxi Botanical Garden of Medicinal Plants, Nanning, China; ^4^Faculty of Veterinary and Animal Sciences, The Islamia University of Bahawalpur, Bahawalpur, Pakistan

**Keywords:** oxidative stress, radix paeoniae alba polysaccharide, amplicon sequencing, gut microbiota, LPS

## Abstract

Accumulating evidence indicated that oxidative stress is closely related to inflammation and the progression of multiple chronic diseases, which seriously threaten the host health. Currently, multiple plant-derived polysaccharides have been demonstrated to ameliorate the negative effects of oxidative stress on the host, but the potential protective effect of radix paeoniae alba polysaccharide (RPAP) on host have not been well characterized. Here, we investigated whether different doses of RPAP administration could alleviate lipopolysaccharide (LPS)-induced intestinal injury and gut microbial dysbiosis in mice. Results indicated that RPAP administration effectively alleviated LPS-induced intestinal damage in dose dependent. Additionally, amplicon sequencing showed that RPAP administration reversed the significant decrease in gut microbial diversity caused by LPS exposure and restored the alpha-diversity indices to normal levels. Microbial taxonomic investigation also indicated that LPS exposure resulted in significant changes in the gut microbial composition, characterized by a decrease in the abundances of beneficial bacteria (*Lactobacillus*, *Alistipes*, *Bacillus*, *Rikenellaceae_RC9_gut_group*, etc.) and an increase in the contents of pathogenic bacteria (*Klebsiella*, *Helicobacter*, *Enterococcus*, etc.). However, RPAP administration, especially in high doses, could improve the composition of the gut microbiota by altering the abundance of some bacteria. Taken together, this study demonstrated that RPAP administration could ameliorate LPS-induced intestinal injury by regulating gut microbiota. Meanwhile, this also provides the basis for the popularization and application of RPAP and alleviating oxidative stress from the perspective of gut microbiota.

## Introduction

Oxidative stress is a state of imbalance between oxidation and antioxidant defense system in the body, which may cause multiple organ damage and dysfunction (Garcia-Mesa et al., 2016; Liu et al., 2019). Previous studies indicated that enzymatic and non-enzymatic antioxidant systems could eliminate accumulated reactive oxygen species (ROS) to decrease oxidative stress and ensure host health (Singh and Vinayak, 2017; Li B. et al., 2019). However, multiple factors such as drinking, smoking, weaning, environmental change, heavy metal and endotoxin could result in the massive production and accumulation of ROS and thus causing oxidative stress (Xu J. et al., 2020; Caliri et al., 2021). Studies demonstrated that oxidative stress caused by ROS can cause many chronic diseases in humans and animals such as aging, inflammation, atherosclerosis and even cancer (Wigner et al., 2021; Kalinina et al., 2022). Consequently, reducing oxidative stress is essential to ensure human and animal health. Recently, research about gut microbiota has attracted increasing attention due to its important roles in oxidative stress and oxidative stress-related diseases (Han et al., 2021; Hao et al., 2021).

Gut microbiome interacts with the host and plays a key role in host health (Maslowski et al., 2009; Das et al., 2021). Statistically, the normal intestine harbors over 100 trillion of microorganisms including bacteria (98%), fungi (0.1%), viruses, protists and archaea, approximately ten times the total amount of host cells (Li et al., 2021a). Increasing evidence demonstrated that gut microbiota not only aid in nutritional acquisition of host, such as amino acids/vitamins synthesis and food digestion and absorption, but also make significant contributions in intestinal epithelium differentiation, mucosal immunity and intestinal homeostasis (Chen H. L. et al., 2021; Wozniak et al., 2021). Importantly, gut microbiota also involved in intestinal mucosal barrier maintenance and immune defense system development, indicating its crucial roles in immunity and disease resistance (Lau et al., 2016; Yun et al., 2022). However, gut microbial composition, diversity and abundance continuously shifts under the influence of host- and environmental-related factors including age, diet, weather and oxidative stress (Makki et al., 2018; Li et al., 2022). Earlier studies demonstrated that stabilized gut microbiota could ensure intestinal functions to function properly, whereas significant changes in gut microbial composition and diversity namely gut microbial dysbiosis may result in etiopathology consequences (Qin et al., 2012; Chi et al., 2019). Besides the common gastrointestinal diseases including diarrhea and colitis, disordered gut microbial community and its related metabolites can also affect both near and far organ systems through blood circulation, causing systemic effects (Chassaing et al., 2015; Li Y. et al., 2019). Notably, gut microbial community or microbial signals can also activate intestinal epithelial cell and other cell types to produce physiological ROS that participate in multiple cellular signaling (Wu et al., 2022). However, gut microbial dysbiosis causes over-accumulation of ROS and oxidative stress. Consequently, maintaining gut microbial homeostasis is critical for alleviating oxidative stress and decreasing oxidative stress-related diseases.

Exogenous antioxidants administration has long been considered as an effective way to relieve oxidative stress and prevent oxidant-related diseases (Chen L. et al., 2021). As an important source of functional dietary antioxidant ingredients, polysaccharides derived from plants, animals and microorganisms have been demonstrated to be essential candidates for developing the promising non-toxic antioxidants (Chen et al., 2022). Among plant polysaccharides, radix paeoniae alba polysaccharide (RPAP) has attracted increasing attention because of its multiple health benefits to the host. RPAP is one of the important active components of radix paeoniae alba, which showed multiple important biological activities such as anti-depression, immunological regulation, whitening and alleviating hepatic injury (Zhang et al., 2020). Moreover, recent reports on RPAP also revealed its key roles in the gastrointestinal disease and relieve oxidative stress. For instance, Zhang et al. (2020) demonstrated that RPAP not only possessed DPPH scavenging activity and reducing power but also could alleviate H_2_O_2_-induced oxidative damage in PC12 cells. Although numerous investigations indicated the positive regulatory effects of RPAP on the host health, it remains unclear whether RPAP could ameliorate intestinal injury and gut microbial dysbiosis caused by LPS. Here, we explored the protective effect of RPAP on LPS induced oxidative damages of intestine.

## Materials and methods

### Animal experiments and sample collection

A group of 100 28-day-old healthy Kunming mice (initial weight 25 ± 2 g) were purchased and maintained under the standard ambient temperature, sanitary condition and illumination as previously described. Prior to the experiment, all the subjects were performed physical examinations to avoid deformity and other congenital diseases. After acclimatization for 3 days, an equal number of mice (*n* = 20, 10 male and 10 female) were divided into control group, LPS-induced group (LPS), low dose RPAP administration group (75 mg/Kg, RL), medium dose RPAP administration group (150 mg/Kg, RM) and high dose RPAP administration group (300 mg/Kg, RH). Mice in RPAP administration groups were compulsively gavaged with 0.2 ml of RPAP with different dose, whereas the mice in control and LPS group received same dose of normal saline by the same way from day 1 to 21. Throughout the trial, all the mice were provided sufficient feed and water. Mice in the LPS, RH, RM and RL groups were intraperitoneally injected with LPS (10 mg/kg) but controls were injected with the same dose of normal saline on days 22 of the experimental study. After 12 h, all mice were dissected and jejunum, ileum, colon and cecum were collected. Subsequently, the sterile fecal samplers were applied to collect rectal feces of each subjects. The achieved samples were immediately sub-sampled from the intermediate region to maximally decrease pollution by bedding and flooring and then snap-frozen utilizing liquid nitrogen and stored at –80°C for further study.

### Histological observations

The specific procedures and processes for tissue section preparation and H&E staining were based on previous studies. Briefly, fresh tissues acquired from dissected mice such as jejunum, ileum, cecum and colon were fixed in 4% paraformaldehyde, dehydrated in gradient ethanol and xylene, embedded in paraffin and prepared 4–5 μm sections. The tissue sections were subsequently subjected to stain with hematoxylin. Representative micrographs of histological sections were taken using inverted microscope.

### DNA extraction and illumina MiSeq sequencing

All frozen colonic contents were thawed and homogenized and then subjected to genomic DNA extraction using QIAamp DNA Mini Kit (QIAGEN, Hilden, Germany) following suggested instructions of manufacturer. Subsequently, the extracts were performed electrophoresis and quantification to evaluate their concentration and integrity. To characterize gut microbial composition and changes, we amplified the V3/V4 region using specific primers (338F: ACTCCTACGGGAGGCAGCA and 806R: GGACTACHVGGGTWTCTAAT) synthesized based on conserved regions. The specific procedures of the PCR amplification was determined based on previous study (Hu et al., 2016). Purification and fluorescent quantitation of amplified products were conducted to prepare sequencing library. The initial libraries need to be properly modified including sequence end repair, sequencing libraries enrichment and purification. Qualified libraries with a concentration greater than 2 and only one peak were performed paired-end sequencing using MiSeq sequencing machine. To obtain more accurate data, we performed further processing on the initial data from sequencing. The short (less than 200 bp), mismatched, unqualified and chimera sequences were abandoned to acquire effective sequence. According to 97% sequence similarity, the qualified sequences were clustered and OTUs partitioned. Alpha diversity indices were calculated based on the quantity of OTUs in each sample to explore the changes in gut microbial diversity and abundance. Meanwhile, PCoA plots were also generated to visualize the differences in gut microbial principal components. Metastats analysis and LEfSe were used to recognize differential taxon. Statistical analysis of data was performed using R (v3.0.3) and GraphPad Prism (version 8.0c). *p*-values (means ± SD) < 0.05 were considered statistically significant.

## Results

### Histological observations

**Figure 1:**
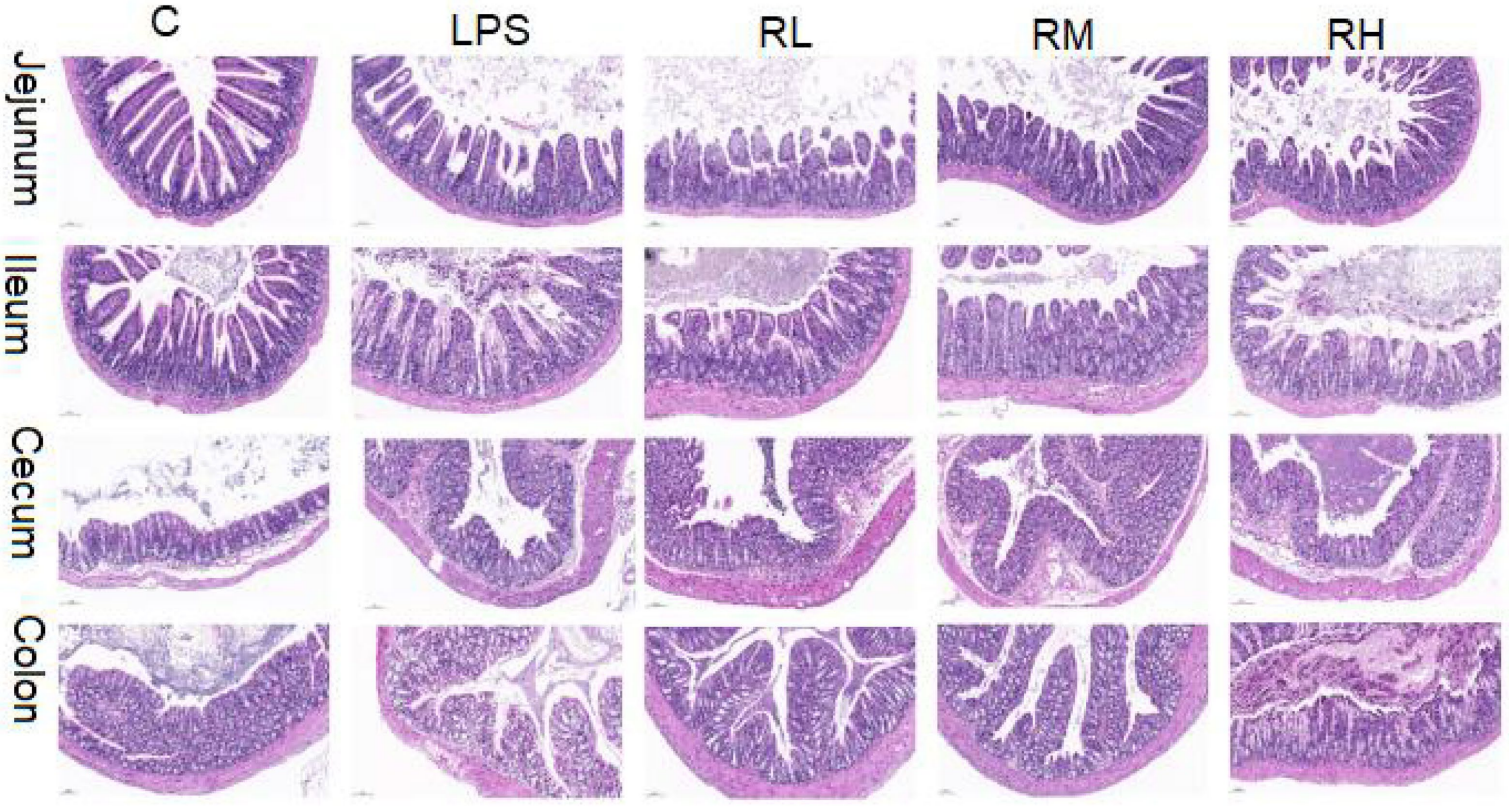
Radix paeoniae alba polysaccharide (RPAP) administration ameliorated LPS-induced intestinal injury in mice. Histological examination of the jejunum, ileum, caecum, and colon.

The histopathological alterations in jejunum, ileum, cecum and colon are presented in Figure 1. H&E staining revealed that the intestines in control group were integrated with clear borders, whereas those in LPS-induced mice were arranged loosely, irregularly and disorderly, accompanied by mucosal edema and infiltration of inflammatory cells in some tissues. However, RPAP administration can alleviate the damage of intestinal villi caused by LPS, indicating that RPAP has a protective effect on LPS-induced intestinal oxidative damage.

### Data acquisition and analysis

**Figure 2:**
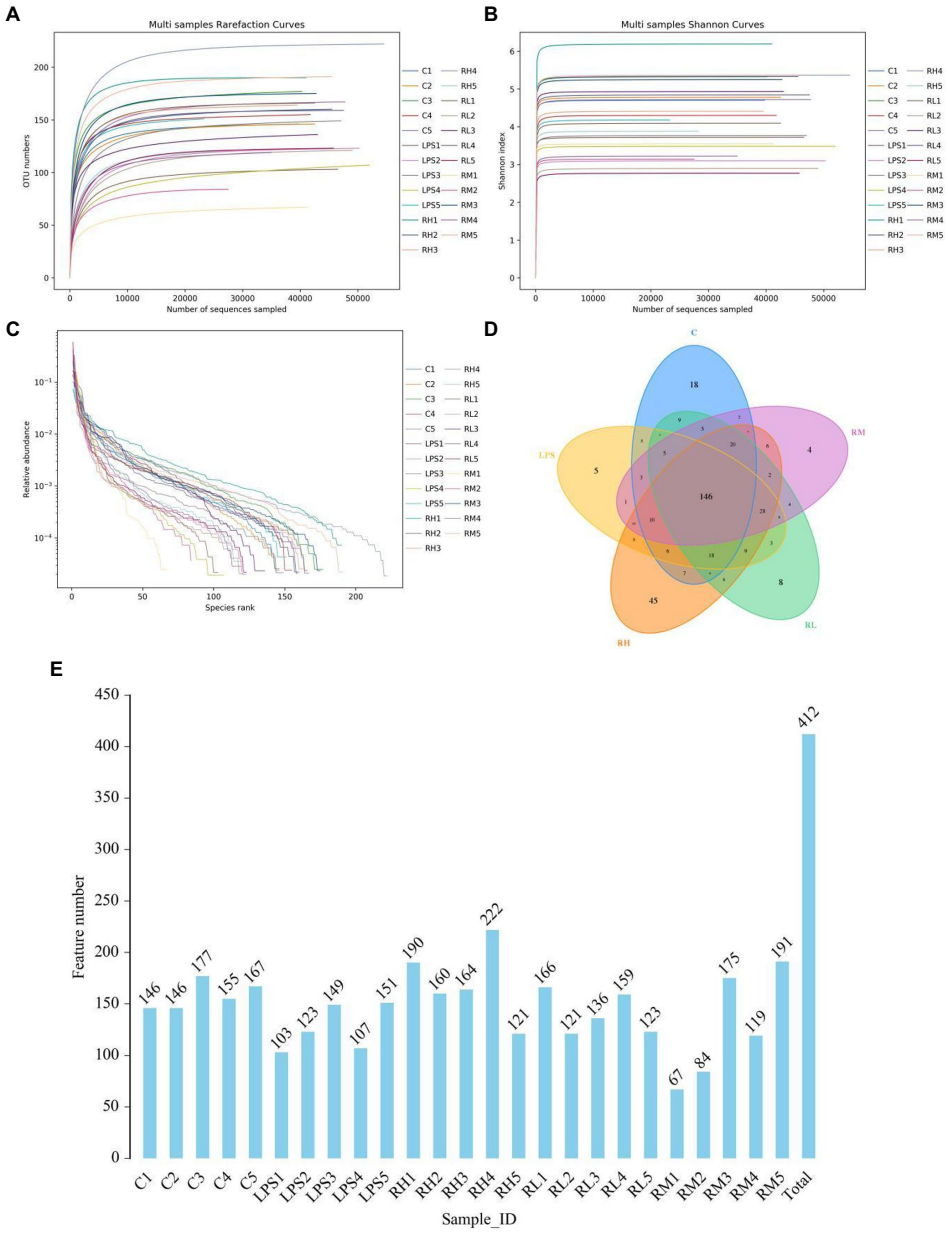
Operational taxonomic units (OTUs) distribution and feasibility analysis of data. Rarefaction **(A,B)** and rank abundance curves **(C)** were applied for assessing the depth of amplicon sequencing. **(D)** The numbers in the different colored areas represent the quantity of OTUs. **(E)** Histogram shows the number of OTUs in each sample.

In this study, 30 samples from five groups were performed amplicon sequencing to explore the protective effect of RPAP on the gut microbiota. Amplicon sequencing results showed that a total of 44,362 (C = 428,534, LPS = 315,828, RH = 428,534, RM = 428,534 and RL = 428,534) raw sequences were generated (Table 1). Moreover, 428,534 (C = 428,534, LPS = 315,828, RH = 428,534, RM = 428,534 and RL = 428,534) valid sequences were totally acquired after quality evaluation, with an effective rate of more than 60%. The multi samples rarefaction and Shannon curves of all samples showed the sufficient species coverage (Figures 2A,B). Meanwhile, the species rank curve in each sample was wide and decreased slowly, showing the satisfactory sequencing evenness and richness (Figure 2C). The effective sequences were subsequently clustered into 100 OTUs, ranging from 200 to 330 OTUs per sample, based on 97% nucleotide-sequence similarity (Figures 2D,E). Moreover, the amount of unique OTUs in the C, LPS, RH, RM and RL groups were 100, 200, 300, 104, and 22, respectively and 169 OTUs were in common, accounting for 93.02% of the overall OTUs.

**Table 1:** The bacterial sequence information of each sample.

Sample	Raw reads	Clean reads	Denoised reads	Merged reads	Effective reads	Effective (%)
C1	68,036	52,257	52,087	48,704	40,403	59.38
C2	77,432	58,282	57,999	53,401	43,243	55.84
C3	56,284	42,172	42,148	41,602	41,351	73.46
C4	58,947	46,044	45,966	44,992	42,555	72.19
C5	69,729	53,398	53,284	51,678	48,621	69.72
LPS1	64,636	49,059	49,015	48,549	46,717	72.27
LPS2	64,194	51,745	51,715	51,426	50,804	79.14
LPS3	62,440	48,954	48,915	48,498	47,596	76.22
LPS4	76,382	57,735	57,695	56,859	52,419	68.62
LPS5	34,165	25,663	25,603	24,842	23,775	69.58
RL1	81,274	52,405	52,232	48,378	43,312	53.29
RL2	63,440	50,110	50,090	49,780	49,504	78.03
RL3	75,268	54,901	54,741	50,820	43,270	57.48
RL4	69,318	53,425	53,320	51,681	48,231	69.57
RL5	75,682	50,548	50,495	49,256	46,207	61.05
RM1	75,582	56,909	56,864	54,392	41,475	54.87
RM2	46,053	34,584	34,505	32,764	27,850	60.47
RM3	71,199	53,963	53,704	50,390	43,309	60.82
RM4	54,813	42,196	42,082	40,011	35,407	64.59
RM5	79,187	57,186	56,923	52,672	46,506	58.72
RH1	82,728	63,680	63,241	54,383	42,392	51.24
RH2	66,431	51,130	50,986	48,987	46,180	69.51
RH3	57,919	41,715	41,559	40,869	40,432	69.8
RH4	77,139	57,122	57,077	56,330	55,834	72.38
RH5	37,160	29,324	29,309	28,959	28,907	77.79

### Radix paeoniae alba polysaccharide administration significantly altered the gut microbial diversity

**Figure 3:**
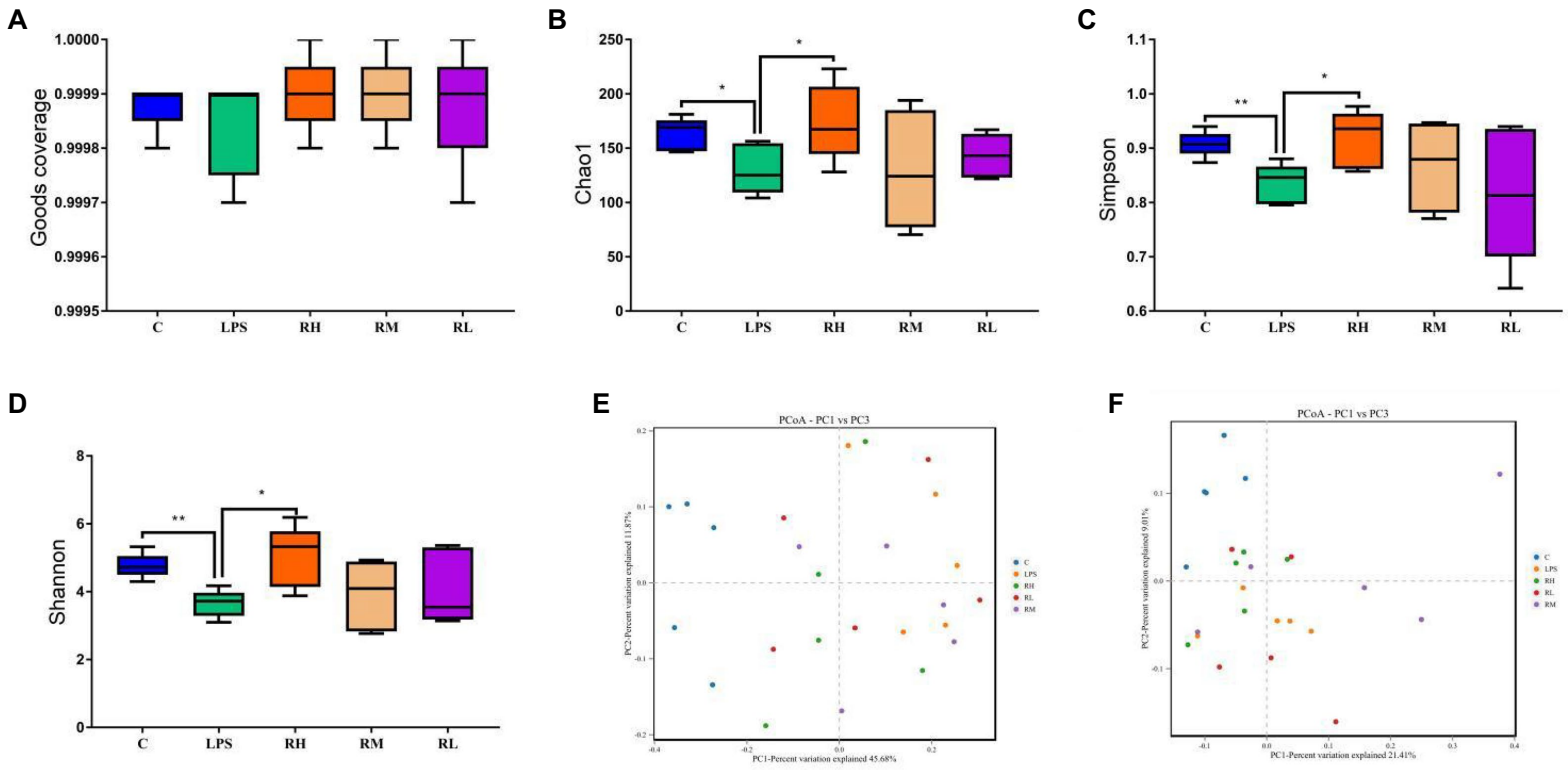
Radix paeoniae alba polysaccharide (RPAP) administration restored significant decrease in gut microbial diversity caused by LPS exposure. The differences of gut microbial alpha-diversity among C, LPS, RH, RM, and RL groups were computed by the Good’s coverage **(A)**, Chao1 **(B)**, ACE **(C)** and Shannon **(D)** indices. **(E,F)** PCoA plots based on the weighted and unweighted UniFrac distance. All data was represented as mean ± SD. ^*^p < 0.05, ^**^p < 0.01.

To further assess the regulating effect of RPAP in gut microbiota, we comparatively analyzed changes in gut microbial diversities between different groups. Good’s coverage estimations of each group ranged from 99.85 to 99.93%, demonstrating almost all bacterial phenotypes were identified (Figure 3A). Statistical analysis of alpha diversity showed that there were statistically distinct differences in the Chao1 (288.04 ± 49.86 vs. 190.54 ± 24.21, *p* < 0.01), ACE (287.89 ± 49.89 vs. 190.34 ± 24.11, *p* < 0.01) and Shannon (6.33 ± 0.70 vs. 5.43 ± 0.75, *p* < 0.05) indices between the C and LPS groups, indicating that LPS administration dramatically decrease gut microbial diversity and abundance (Figures 3B–D). However, RPAP administration could reverse LPS-induced gut microbial dysbiosis and its effect was proportional to the RPAP concentration. Specifically, high-dose RPAP administration could restore the gut microbial diversity and abundance to normal levels and significantly higher than those in the LPS group. Moreover, beta diversity analysis revealed that the dots in C and LPS groups were separated but closer together with the RPAP-treated groups, suggesting that LPS significantly affect the gut microbial structure, whereas RPAP administration could alleviate the present phenomenon (Figures 3E,F).

### Comparative analysis of the gut microbial composition between different groups

**Figure 4:**
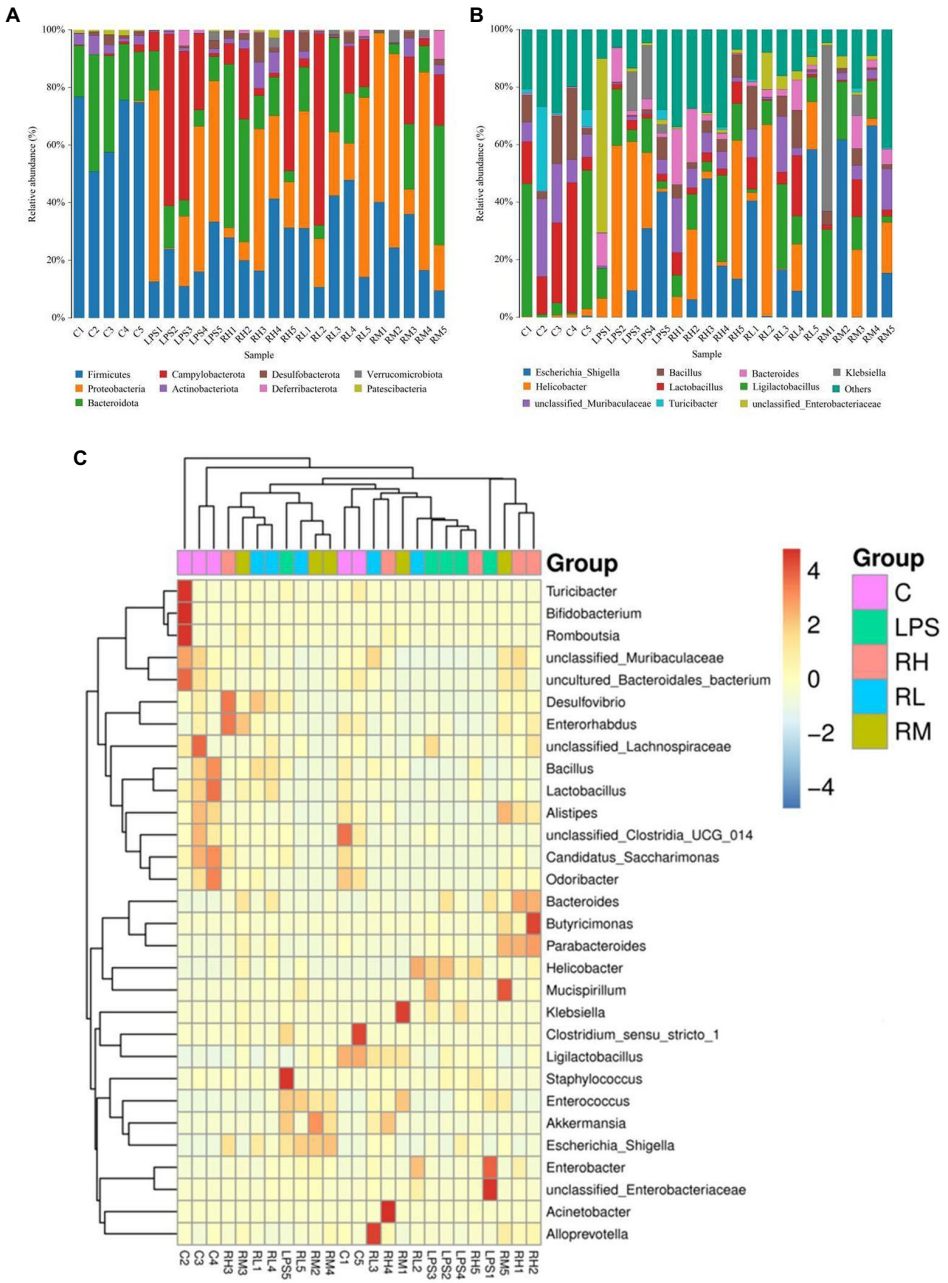
Effects of RPAP administration on gut microbial composition in LPS-induced mice. Composition and relative proportion of dominant bacteria at the phylum **(A)** and genus levels **(B)**. **(C)** Heatmap of the 50 most abundant bacterial genera in the C, LPS, RH, RM, and RL groups.

We also investigated the composition and abundance of dominant intestinal bacteria in different taxonomical levels and observed considerable variability. Specifically, there were 9 phyla and 119 genera found in 30 samples, ranging from 5 to 7 phyla per sample. The phyla *Firmicutes* (67.32, 28.02%) and *Bacteroidota* (25.48, 28.00%) were abundantly present in the C and RH groups, whereas the dominant phyla observed in the RM and RL groups were *Firmicutes* (25.47, 29.03%) and *Proteobacteria* (40.44, 30.55%; Figure 4A). Moreover, the gut microbiota in LPS group was predominated by *Proteobacteria* (36.59%), *Campylobacterota* (32.55%) and *Firmicutes* (17.91%) in descending order. Other phyla such as *Deferribacterota* (0.032, 1.53%, 0.34, 2.46, 0.70%), *Verrucomicrobiota* (0.0075, 0.33, 0.88, 1.08, and 0.32%) and *Patescibacteria* (1.09, 0.066, 0.92, 0.075, and 0.10%) in the C, LPS, RH, RM and RL groups were identified in low abundances. Among recognized genera, *Escherichia_Shigella* (13.96, 16.95, and 24.38%), *Helicobacter* (32.54, 14.03, and 21.62%) and *Ligilactobacillus* (10.72, 14.34, and 11.32%) were the most predominant genus in the LPS, RH and RL groups, accounting for over 45% of the total composition (Figure 4B). Moreover, the preponderant bacterial genus found in gut microbiota in the C group were *Ligilactobacillus* (20.61%), *Lactobacillus* (20.60%) and *unclassified_Muribaculaceae* (13.98%), whereas *Escherichia_Shigella* (24.72%) was the most dominant genus in the RM group, followed by *Ligilactobacillus* (14.84%) and *Klebsiella* (13.95%). Moreover, gut microbial distribution and variability between the C, LPS, RH, RM, and RL groups could also be observed by the visualized clustering heatmap (Figure 4C).

**Figure 5:**
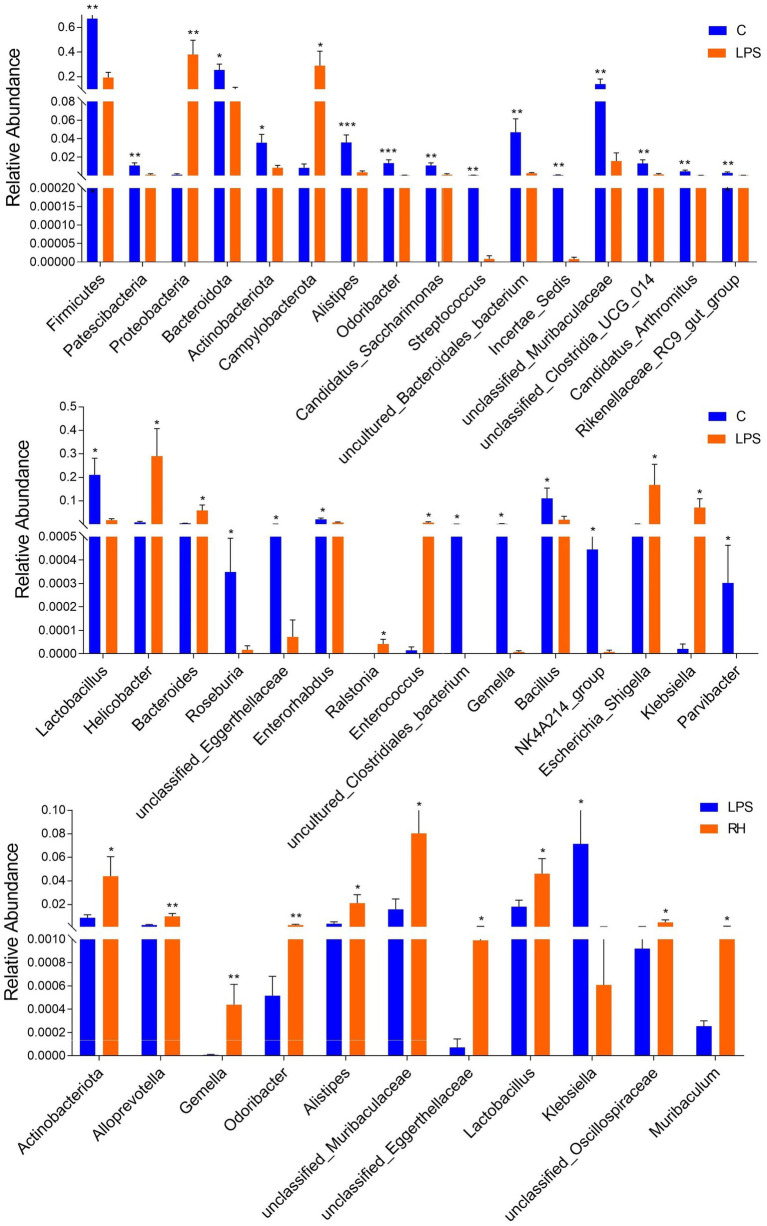
Statistical analysis of differential bacteria between different groups at the phylum and genus levels. All data was represented as mean ± SD. ^*^p < 0.05, ^**^p < 0.01.

**Figure 6:**
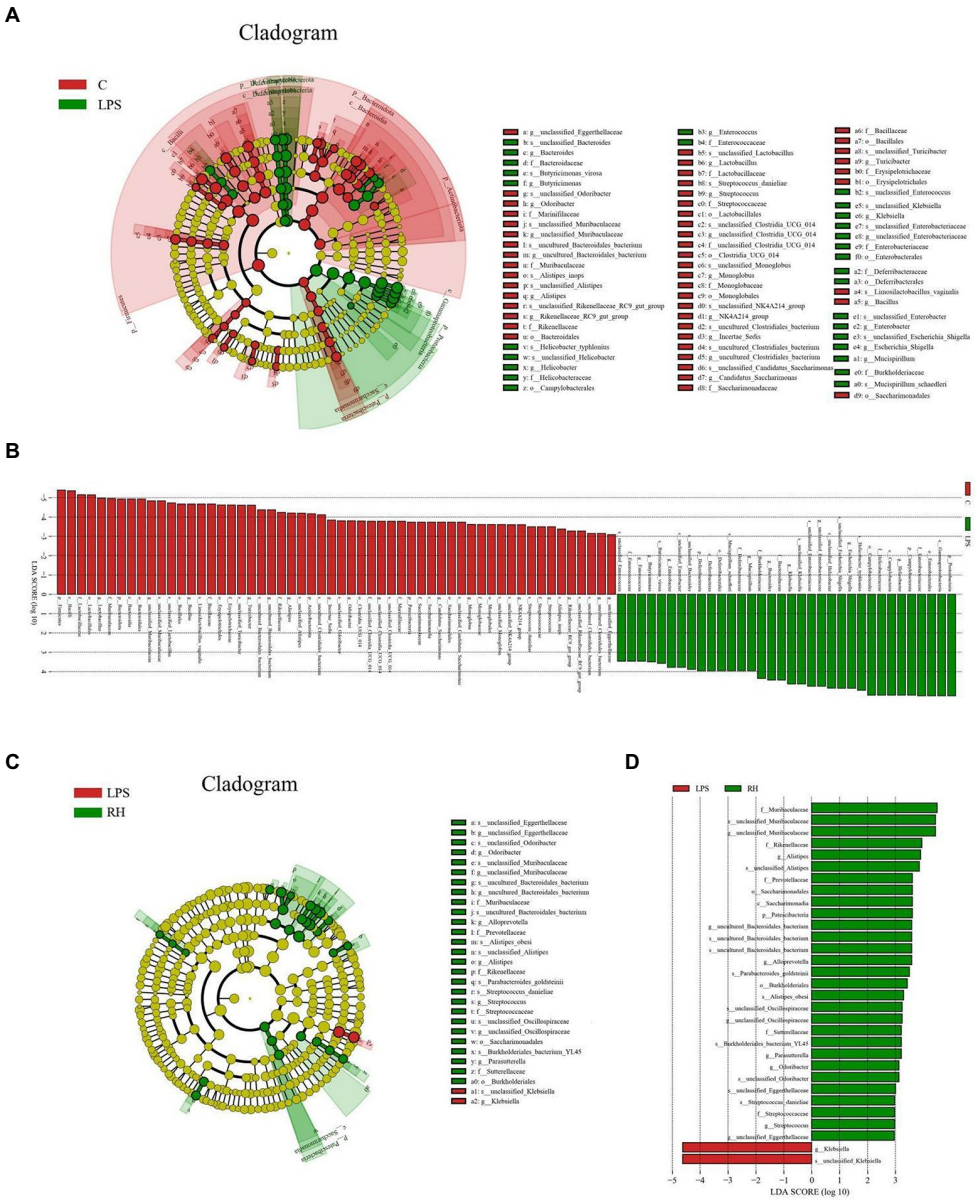
Linear discriminant analysis effect size (LEfSe) integrated with Linear discriminant analysis (LDA) scores recognized differentially abundant taxon associated with RPAP administration. **(A,C)** Cladogram shows the phylogenetic distribution of differential biomarkers. **(B,D)** The criterion for significant difference is LDA scores > 2.

To further explore the influence of RPAP on gut microbial composition, Metastats analysis was performed for different classification levels. At the phylum level, the LPS group showed dramatically higher abundance of *Proteobacteria* and *Campylobacterota*, whereas the C group enriched for *Firmicutes*, *Patescibacteria*, *Bacteroidota*, and *Actinobacteriota* (Figure 5). Compared with the C group, the gut microbiota in the LPS group showed distinct decrease in the relative abundance of *Alistipes*, *Odoribacter*, *Candidatus_Saccharimonas*, *Streptococcus*, *uncultured_Bacteroidales_bacterium*, *Incertae_Sedis*, *unclassified_Muribaculaceae*, *unclassified_Clostridia_UCG_014*, *Candidatus_Arthromitus*, *Rikenellaceae_RC9_gut_group*, *Lactobacillus*, *Roseburia*, *unclassified_Eggerthellaceae*, *Enterorhabdus*, *uncultured_Clostridiales_bacterium*, *Gemella*, *Bacillus*, *NK4A214_group*, and *Parvibacter*, whereas *Helicobacter*, *Bacteroides*, *Ralstonia*, *Enterococcus*, *Escherichia_Shigella* and *Klebsiella* increased significantly. Furthermore, a comparison of the LPS and RH groups showed significant decline in the abundance of 9 genus (*Alloprevotella*, *Gemella*, *Odoribacter*, *Alistipes*, *unclassified_Muribaculaceae*, *unclassified_Eggerthellaceae*, *Lactobacillus*, *unclassified_Oscillospiraceae* and *Muribaculum*) as well as a significant increase in the abundance of 1 genus (*Klebsiella*). We also observed that six bacterial genera were dramatically different between the LPS and RM groups. Among differential taxa, *Gemella*, *Streptococcus*, *Alloprevotella* and *Muribaculum* were observed to be more abundant in the RM group than in the LPS group, whereas the *Erysipelatoclostridium* and *Lachnospiraceae_NK4A136_group* exhibited the opposite trend (Supplementary Figure S1). The RL group showed dramatically higher abundance of *Oscillibacter* than that of LPS, whereas the relative abundance of *Erysipelatoclostridium* and *Klebsiella* were lower. Considering this discriminant analysis could not detect all the taxon, LEfSe combined with LDA scores were used to further recognize the differential bacteria. Besides the above-mentioned differential taxa, we also observed that *Deferribacterota*, *Mucispirillum*, *Enterobacter* and *Butyricimonas* were significantly more preponderant in LPS group than in the C group, whereas the abundance of *Monoglobus* and *Turicibacter* were lower (Figures 6A,B). For the comparison between the LPS and RH groups, the RH group showed the higher abundance of *Patescibacteria*, *Alistipes_obesi*, *Parasutterella*, and *Streptococcus* (Figures 6C,D).

### Correlation network analysis

**Figure 7:**
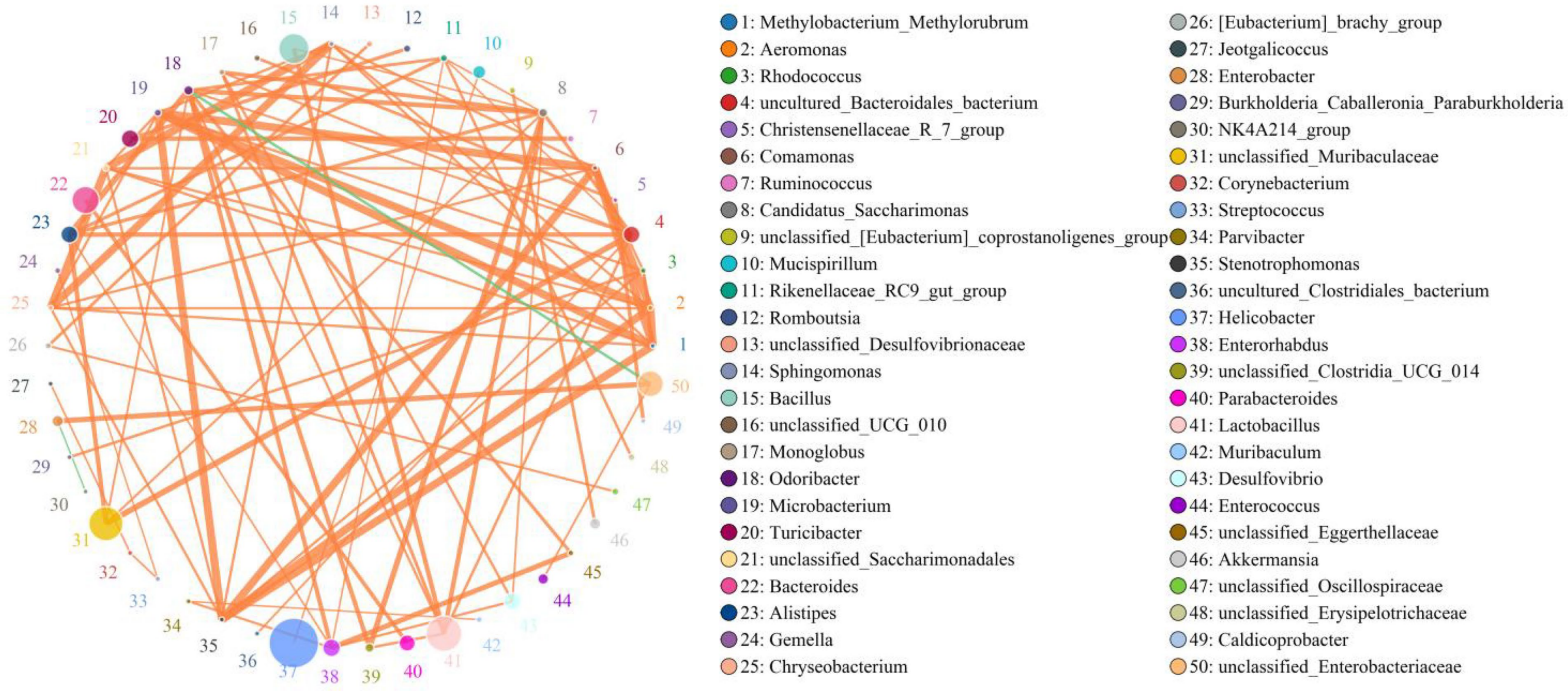
Correlation network analysis of gut bacterial community. Bacterial genera are indicated by differently colored dots. Positive and negative correlations are represented by red and green lines, respectively. Detailed data were shown in the Supplementary Table S1.

*Lactobacillus* was positively associated with *Bacillus* (0.8292), *Rikenellaceae_RC9_gut_group* (0.5239), *Alistipes* (0.6485), *Odoribacter* (0.7208) and *Monoglobus* (0.7562) but negatively related to *Enterococcus* (0.6054) and *Enterobacter* (0.4702; Figure 7). *Bacillus* was positively associated with *Alistipes* (0.5415). *Rikenellaceae_RC9_gut_group* was positively related to *Muribaculum* (0.5190), *Monoglobus* (0.4841), *Erysipelatoclostridium* (0.4410) and *Incertae_Sedis* (0.5865). *Alistipes* was positively associated with *Alloprevotella* (0.4192), *Adlercreutzia* (0.4017), *Parvibacter* (0.5141), *Caldicoprobacter* (0.3976) and *Anaerofustis* (0.4844) but negatively related to *Enterobacter* (0.4419) and *Enterococcus* (0.5266). *Klebsiella* was negatively related to *Alistipes* (0.4264). *Enterococcus* was negatively associated with *Rikenellaceae_RC9_gut_group* (0.6712), *Erysipelatoclostridium* (0.5269), *Incertae_Sedis* (0.4001), *Odoribacter* (0.6703), *Candidatus_Saccharimonas* (0.5884) and *Anaerofustis* (0.5099).

## Discussion

Oxidative stress has been demonstrated to cause septicemia, acidosis, enteritis, pneumonia and decrease meat quality and fertility (Kuwano et al., 2003; Xu W. et al., 2020). As the organ most susceptible to oxidative stress, oxidative stress in the intestinal system can affect the digestion and absorption of nutrients and cause abnormal glandular secretion, intestinal damage, gastrointestinal dysfunction and intestinal infection, seriously threatening animal production and health (Qiao et al., 2013). Gut microbiota is closely related to gut health, thus the gut microbial homeostasis is inevitably affected by intestinal damage caused by oxidative stress (Zhao et al., 2018). Additionally, gut microbial dysbiosis can further affect intestinal permeability, intestinal mucosal immunity and even other organs such as liver and brain, causing more serious pathological consequences (Boursier et al., 2016). Consequently, maintaining the intestinal health and gut microbial homeostasis is critical for host health. As an effective extract of traditional Chinese medicine, polysaccharides have been demonstrated to exhibit multiple pharmacological effects such as anti-inflammatory, anti-tumor, antioxidant and hypoglycemic, but there are still few studies on RPAP. Here, we systematically explored the protective effects of RPAP administration on intestinal health and microbiota in LPS-induced mice.

Gut microbial diversity has long been regarded as an important indicator for assessing gut microbial homeostasis (Jin et al., 2019). Typically, gut microbial community is in a relatively stable status and this is the prerequisite for intestine to perform various complex physiological functions (Li et al., 2021b). Research indicated that many factors such as gender, age and diet could cause dynamic changes in gut microbiota but this physiological fluctuation cannot destroy intestinal homeostasis and host health (Jiao et al., 2016; Dias et al., 2018). However, strong external stimulus such as antibiotic, oxidative damage, malignant tumor and heavy metal may impair intestinal morphology and environment, which in turn affect microbial growth and survival and force existing microbes to adapt to new environment, causing gut microbial dysbiosis (Lin et al., 2021; Liao et al., 2022). Xu et al. (2019) reported that LPS exposure impaired intestinal morphology and resulted in gut microbial dysbiosis in mice. Moreover, the disruptive effect of LPS on gut microbial homeostasis was also demonstrated in other exploratory experiments (Li et al., 2020; Go et al., 2021). Consistent with previous investigations, we observed that LPS exposure resulted in a significant decrease in the gut microbial alpha-diversity, accompanied by intestinal damage. However, RPAP supplementation increased the gut microbial diversity indices and was positively correlated with its concentration, indicating that RPAP could restore LPS-induced gut microbial dysbiosis. Currently, the decreased gut microbial diversity has been demonstrated to be strongly associated with the progression of multiple chronic diseases such as diabetes, obesity and nonalcoholic fatty liver disease (Musso et al., 2011; Yu et al., 2016). As an important biomarker of gut microbial dysbiosis, decreased gut microbial diversity may impair intestinal barrier function and mucosal immune system, causing severe gastrointestinal infection, diarrhea and colonitis (Koh et al., 2015; Jin et al., 2018). Furthermore, gut microbial dysbiosis also cause pathogenic bacteria and toxic metabolites more easily cross the intestinal barrier and extend its adverse effects beyond the gastrointestinal system, causing systemic effects (Sekirov et al., 2010). Gut microbial beta-diversity is also an important tool for assessing gut microbiota. We observed that the individuals of the C group were clustered together but separated from the LPS group, indicating significant changes in the gut microbial main components. However, the gut microbial main components of the RH group were closer to the C group compared with the LPS group, suggesting the improvement in the gut microbial structure.

Consistent with previous investigations, this study also indicated that LPS exposure caused distinct shifts in gut microbial composition and structure, perturbing gut microbial homeostasis. However, RPAP administration can reverse this phenomenon and result in changes in some functional bacteria, such as *Alloprevotella*, *Lactobacillus*, *Alistipes*, *Bacillus* and *Rikenellaceae_RC9_gut_group*, which may play vital roles in intestinal ecosystem and health. *Alloprevotella* has been reported to produce acetic acid and succinic acid, which contributed to maintaining gut microbial homeostasis and decreasing the risk of lifetime cardiovascular disease (Yuan et al., 2021). Numerous investigations revealed that *Lactobacillus* possessed important biological properties such as secreting antimicrobial substance, improving the intestinal environment, maintaining intestinal microbial homeostasis (Maroju et al., 2021; Stivala et al., 2021). Besides the above-mentioned characteristics, *Lactobacillus* also contribute to controlling endotoxin, improving immunity, food digestibility and biological potency, indicating its key roles in host growth and health (Gao et al., 2018; Veisi et al., 2022). *Alistipes* has been demonstrated to be the potential producer of short-chain fatty acids (SCFAs) (Khan et al., 2020). As the acknowledged intestinal beneficial metabolites, SCFAs have been demonstrated to function against pathogens, decreasing oxidative stress, maintaining gut microbial balance, and regulating the intestinal barrier (Elamin et al., 2013; Pekmez et al., 2019). Furthermore, SCFAs also participated in the positive regulation of the host metabolism, cell proliferation and immunological function (Van Rymenant et al., 2017; Menni and Valdes, 2019). Recently published research also demonstrated their crucial roles in lowering cholesterol, alleviating intestinal inflammation, reducing diabetes and angiocardiopathy (Thandapilly et al., 2018). Several studies involving *Rikenellaceae* have demonstrated its important roles in degrading plant derived polysaccharide and alleviate inflammation. As a common intestinal probiotic, *Bacillus* is widely used in industry, agriculture and pharmaceutical production. Research indicated that the antibacterial substances produced by *Bacillus* have broad-spectrum bactericidal activity and have strong bactericidal effects on many food-related bacteria (Jones and Knight, 2012). Moreover, *Bacillus* could produce beneficial metabolites such as vitamins, showing great potential in alleviating the gut microbial dysbiosis and treating bacterial infections (Li Y. T. et al., 2019; Wang et al., 2021). In this study, we also found that LPS exposure resulted in a significant increase in some pathogenic bacteria such as *Klebsiella*, *Helicobacter* and *Enterococcus*. *Klebsiella*, a potential intestinal pathogen, is closely related to multiple diseases such as mastitis, pneumonia, septicemia and hysteritis (Bratu et al., 2005). Early surveys indicated that *Helicobacter* could result in peptic ulcer, gastritis and even lymphoproliferative gastric lymphoma, seriously threatening the host health (Castano-Rodriguez et al., 2017). Notably, some diseases caused by *Helicobacter* may eventually progress to gastric cancer that is one of the most common malignancies worldwide and causes approximately 160,000 deaths annually in China (Choi et al., 2018). *Enterococcus* was previously reported to cause life-threatening cardioperiostitis, sepsis and meningitis (Knoll et al., 2013). Moreover, many antibiotics cannot effectively treat *Enterococcus* infection due to inherent and acquired resistance (Lebreton et al., 2011). Interestingly, RPAP administration reduces the abundance of these potential pathogenic bacteria, which is beneficial to maintain gut microbial homeostasis and protect host health.

## Conclusion

In summary, this study investigated the protective effect of RPAP administration on intestinal health and microbiota in LPS-induced mice. Results indicated that RPAP administration could restore LPS-induced intestinal injury and gut microbial dysbiosis and its effect was positively correlated with concentration. This study filled a gap in the effect of RPAP on the intestinal health and microbial homeostasis caused by LPS and demonstrated that the maintenance of gut microbial balance may be one of the important pathways for RPAP to exert its pharmacological effects. Moreover, these findings also expand the understanding of the health benefits of RPAP and provide a theoretical basis for the development of RPAP products to decrease oxidative stress. However, this study also has some limitations including relatively small sample size and lack of intestinal metabolism experiments.

## Data availability statement

The datasets presented in this study can be found in online repositories. The names of the repository/repositories and accession number(s) can be found at: https://www.ncbi.nlm.nih.gov/, PRJNA875596.

## Ethics statement

The animal study was reviewed and approved by the Animal Welfare and Ethics Committee of Nanjing Agricultural University.

## Author contributions

KL and AL provided the research idea. TS, KL, YW, and JD contributed reagents, materials, and analysis tools. AL wrote the manuscript. MI, MK, KW, YL, and FW revised the manuscript. All authors contributed to the article and approved the submitted version.

## Funding

The study was supported by Start-up fund of Nanjing Agricultural University (804131) and Guangxi Science and Technology Project (GuiKeAD22080012, GuiKeAA18242040, and GuiKeZY20198018).

## Conflict of interest

The authors declare that the research was conducted in the absence of any commercial or financial relationships that could be construed as a potential conflict of interest.

## Publisher’s note

All claims expressed in this article are solely those of the authors and do not necessarily represent those of their affiliated organizations, or those of the publisher, the editors and the reviewers. Any product that may be evaluated in this article, or claim that may be made by its manufacturer, is not guaranteed or endorsed by the publisher.
