# Supplementary material for: Radix paeoniae alba polysaccharide attenuates lipopolysaccharide-induced intestinal injury by regulating gut microbiota
Source: Front Microbiol. 2023 Jan 12;13:1064657. doi: 10.3389/fmicb.2022.1064657 (PMC9878331; doi:10.3389/fmicb.2022.1064657)
Supplement: Supplementary file 1 [file Data_Sheet_1.docx]

**Supplementary Table S1.** Correlation analysis of gut microbial community.

| source | target | weight | correlation |
| --- | --- | --- | --- |
| *Lactobacillus* | *Bacillus*  *Turicibacter*  *Enterorhabdus*  *Desulfovibrio*  *Alistipes*  *Enterobacter*  *Enterococcus*  *Odoribacter*  *Candidatus_Saccharimonas*  *Rikenellaceae_RC9_gut_group*  *Monoglobus*  *Erysipelatoclostridium*  *Corynebacterium*  *Incertae_Sedis*  *Anaerofustis* | 0.8292  0.5265  0.4531  0.5992  0.6485  0.4702  0.6054  0.7208  0.8312  0.5239  0.7562  0.4036  0.4637  0.6395  0.5098 | positive  positive  positive  positive  positive  negative  negative  positive  positive  positive  positive  positive  positive  positive  positive |
| *Bacillus* | *Turicibacter*  *Desulfovibrio*  *Alistipes*  *Mucispirillum*  *Odoribacter*  *Candidatus_Saccharimonas*  *Gemella*  *Monoglobus*  *Corynebacterium*  *Incertae_Sedis*  *Streptococcus*  *Jeotgalicoccus* | 0.4689  0.5546  0.5415  0.3966  0.5392  0.6829  0.4254  0.6612  0.6478  0.4529  0.5239  0.4608 | positive  positive  positive  negative  positive  positive  positive  positive  positive  positive  positive  positive |
| *Alistipes* | *Alloprevotella*  *Enterobacter*  *Enterococcus*  *Odoribacter*  *Candidatus_Saccharimonas*  *Rikenellaceae_RC9_gut_group*  *Muribaculum*  *Monoglobus*  *Incertae_Sedis*  *Adlercreutzia*  *Parvibacter*  *Caldicoprobacter*  *Anaerofustis* | 0.4192  0.4419  0.5266  0.9015  0.7753  0.7651  0.5459  0.5751  0.6065  0.4017  0.5141  0.3976  0.4844 | positive  negative  negative  positive  positive  positive  positive  positive  positive  positive  positive  positive  positive |
| *Rikenellaceae_RC9_gut_group* | *Muribaculum*  *Monoglobus*  *Erysipelatoclostridium*  *Incertae_Sedis*  *Parvibacter*  *Anaerofustis* | 0.519  0.4841  0.441  0.5865  0.4412  0.4826 | positive  positive  positive  positive  positive  positive |
| *Klebsiella* | *Alistipes*  *Staphylococcus* | 0.4264  0.4156 | negative  positive |
| *Enterococcus* | *Odoribacter*  *Candidatus_Saccharimonas*  *Rikenellaceae_RC9_gut_group*  *Erysipelatoclostridium*  *Incertae_Sedis*  *Anaerofustis* | 0.6703  0.5884  0.6712  0.5269  0.4001  0.5099 | negative  negative  negative  negative  negative  negative |


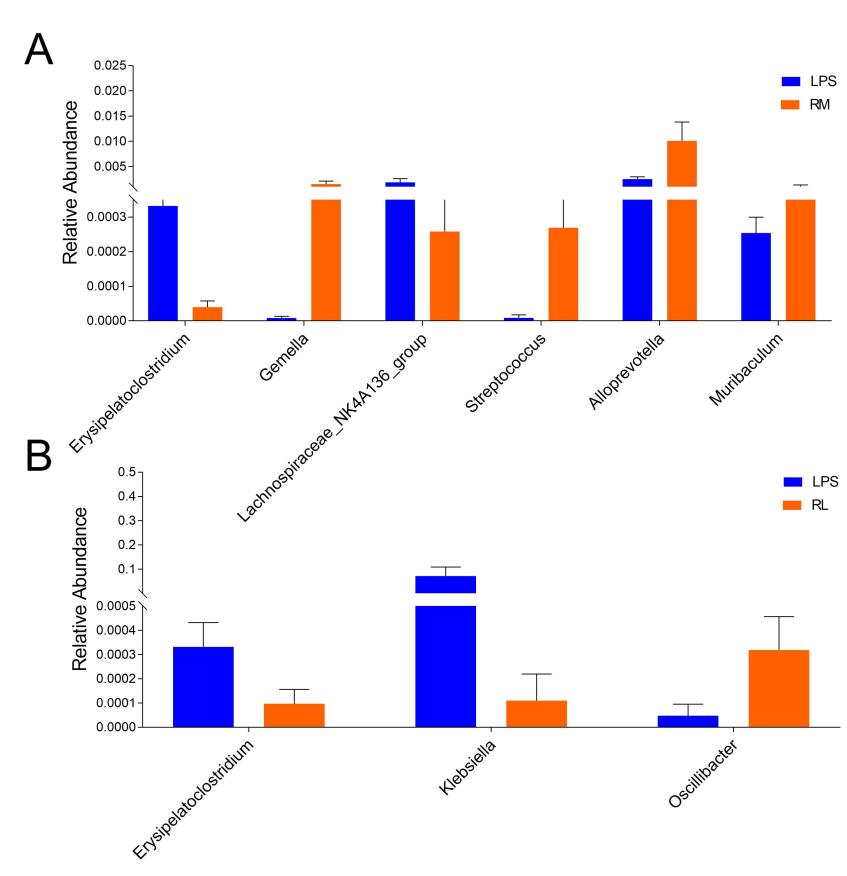


**Supplementary Figure S1.** Statistical analysis of differential bacteria between different groups at the phylum and genus levels. All data was represented as mean ± SD. * p < 0.05, ** p < 0.01.
